# Lack of Effect of Lamivudine on Ebola Virus Replication

**DOI:** 10.3201/eid2103.141862

**Published:** 2015-03

**Authors:** Lisa E. Hensley, Julie Dyall, Gene G. Olinger, Peter B. Jahrling

**Affiliations:** National Institutes of Health, Frederick, Maryland, USA

**Keywords:** Ebola virus disease, Ebola virus, viruses, antiviral drugs, lamivudine, toremifene, virus replication, cell lines, primary human macrophages

**To the Editor:** The unprecedented number of Ebola virus disease (EVD) cases in western Africa has compelled the world to consider experimental and off-label therapeutics to mitigate the current outbreak. For clinicians, approved drugs are an attractive solution because of known safety profiles and availability.

Oral lamivudine (GlaxoSmithKline, Brentford, UK), a US Food and Drug Administration–approved anti-HIV drug, has been suggested as a possible antiviral agent against Ebola virus (EBOV). In September 2014, a Liberian physician, Dr. Gorbee Logan, reported positive results while treating EVD with lamivudine (*1*). Thirteen of 15 patients treated with lamivudine survived presumed EVD and were declared virus free. Clinical confirmation of EVD in these cases remains to be verified.

Our laboratory had previously assessed this antiretroviral compound in drug screens against EBOV and observed no discernable antiviral activity. However, given the recent testimonials regarding lamivudine effectiveness in treating EBOV-infected patients in Africa, we conducted additional studies to determine whether our previous assertion that lamivudine lacked any direct antiviral activity was correct.

Lamivudine is a nucleoside analog reverse transcription inhibitor of HIV and hepatitis B virus that acts as a synthetic cytidine analog. Incorporation of the active triphosphate form into viral DNA results in chain termination. Studies have demonstrated that lamivudine is a weak inhibitor of mammalian α, β, and γ DNA polymerases (*2*). Lamivudine would not be expected to inhibit the replication of a negative-strand RNA virus.

The activity of lamivudine against EBOV infection was evaluated in a cell-based ELISA with 1995 isolate EBOV *H. sapiens*-tc/COD/1995/Kikwit (EBOV/Kik) (*3*). Three cell lines were tested: Vero E6 (African green monkey kidney, ATCC CRL-1586), Hep G2 (human hepatoma, ATCC HB-8065), and human monocyte-derived macrophages. Macrophages were generated by treating CD14^+^ cells for 7 days with macrophage colony–stimulating factor and conditioned medium. Cells were treated with compounds in 3-, 4-, or 8- point dose response curves with 2-fold dilutions starting at 80 µmol/L or 320 µmol/L oral lamivudine. Toremifene (T7204–5MG; Sigma-Aldrich, St. Louis, MO, USA) was used as a positive control for activity against EBOV and tested at 2-fold dilutions starting at 25 µmol/L. One hour after drug addition, the cells were infected at a multiplicity of infection of 0.5 or 1 with EBOV/Kik. Experiments were run on duplicate plates or the entire experiment was run on 2 separate days. At 48 hours after infection, cells were formalin-fixed and stained with a primary antibody against EBOV (antibodies against viral matrix protein or glycoprotein) and a secondary antibody (Alexa-488 or horseradish peroxidase).

No direct antiviral effect for lamivudine was observed at concentrations ≤320 µmol/L in Vero E6 cells (Table). Because optimal efficacy of the drug requires phosphorylation, lack of activity may be caused by poor phosphorylation in Vero E6 cells (*6*). Therefore, we also assessed HepG2 cells and primary human monocyte–derived macrophages sensitive to EBOV infection. Toremifene was included as a positive control. Toremifene is a US Food and Drug Administration–approved drug that was reported to have direct antiviral activity in cell culture and to protect mice infected with mouse-adapted EBOV (*3*). As expected, toremifene inhibited EBOV at low micromolar concentrations (Table).

**Table:** Inhibitory effects of test compounds on Ebola virus replication*

Virus variant or subtype	Cell type	Lamivudine EC_50_, μmol/L†	Toremifene EC_50_ ± SD, μmol/L†
EBOV/Kik	Vero E6	>80	5.7 ± 0.7
EBOV/Kik	Vero E6	>320	12.0 ± 1.0
EBOV/Gue	Vero E6	>320	≈8
EBOV/Kik	HepG2	>80	1.6 ± 0.1
EBOV/Kik	HepG2	>320	5.5 ± 0.1
EBOV/Kik	Macrophages	>320	≈25
EBOV/Kik	Macrophages	>320	18.3 ± 0.8
HIV-1	Macrophages	0.002‡	ND
HIV-1	Monocytes	0.69‡	ND
HIV-1 (multiple subtypes)	PBMC	0.002–2.5‡	ND
HBV	HepG2 (2.2.15)	0.002§	ND

*EBOV, Ebola virus; EC_50_, 50% effective concentration; BMC, peripheral blood mononuclear cells; ND, not done; HBV, hepatitis B virus. †EC_50_s were determined by using an EBOV ELISA with antibodies against glycoprotein or viral matrix protein as described (*3*). ‡Data from Schinazi (*4*). §Data from Kruining et al. (*5*).

Finally, we assessed the antiviral activity of the compounds against a recent isolate prototype from the current outbreak, EBOV *H. sapiens*-tc/GIN/2014/Guéckédou-C05 (EBOV/Gue) to test whether inhibition of EBOV/Gue by lamivudine was different from that of the reference Kikwit strain. In contrast to a known active compound (toremifene), lamivudine showed no direct antiviral activity.

The current data suggest that lamivudine does not directly inhibit EBOV RNA polymerase or replication of the virus. Systemic and off-target effects, while not previously described, might be possible. To address this possibility, we plan to assess lamivudine in the mouse model of EVD and will report these findings when available. However, on the basis of these in vitro tests, there is no foundation for recommending lamivudine for treatment of EVD in human patients.
